# Welfare Assessment in Calves Fattened According to the “Outdoor Veal Calf” Concept and in Conventional Veal Fattening Operations in Switzerland

**DOI:** 10.3390/ani10101810

**Published:** 2020-10-05

**Authors:** Lara Moser, Jens Becker, Gertraud Schüpbach-Regula, Sarah Kiener, Sereina Grieder, Nina Keil, Edna Hillmann, Adrian Steiner, Mireille Meylan

**Affiliations:** 1 Clinic for Ruminants, Vetsuisse-Faculty, University of Bern, 3012 Bern, Switzerland; lara.moser@immerda.ch (L.M.); jens.becker@vetsuisse.unibe.ch (J.B.); sarah.kiener@vetsuisse.unibe.ch (S.K.); adrian.steiner@vetsuisse.unibe.ch (A.S.); 2 Veterinary Public Health Institute, Vetsuisse-Faculty, University of Bern, 3012 Bern, Switzerland; gertraud.schuepbach@vetsuisse.unibe.ch; 3 Ethology and Animal Welfare Unit, Department of Environmental Systems Science, Institute of Agricultural Sciences, Eidgenössische Technische Hochschule (ETH), 8053 Zurich, Switzerland; sereina.grieder@tierschutz.com (S.G.); edna.hillmann@hu-berlin.de (E.H.); 4 Centre for Proper Housing of Ruminants and Pigs, Federal Food Safety and Veterinary Office, Agroscope, 8356 Tänikon, Switzerland; nina.keil@agroscope.admin.ch; 5 Animal Husbandry Division, Albrecht Daniel Thaer-Institute of Agricultural and Horticultural Sciences, Humboldt University of Berlin, 10099 Berlin, Germany

**Keywords:** veal industry, animal welfare, housing, pneumonia, abomasal ulcers, antimicrobial use, treatment incidence

## Abstract

**Simple Summary:**

Antimicrobial use in humans and animals leads to the selection of resistant bacteria, a serious threat to human and animal health, as such bacteria can lead to treatment failure and death. With the “outdoor veal calf” concept, a novel calf fattening system was developed that allows for reducing antimicrobial use by 80% through improvements in management and housing, such as health check before purchase, short transport, vaccination, quarantine in individual hutches, and fattening in small groups in a roofed, straw-bedded paddock with a group hutch for shelter. In that system, veal calves spend their entire lives outdoors in the fresh air. In our study, we wanted to make sure that the observed reduction in antimicrobial treatments was not achieved at the cost of animal welfare, i.e., that sick animals were not left without treatment in order to obtain better figures for treatment reduction. Our results show that calves in the “outdoor veal calf” system had fewer signs of respiratory and digestive diseases than control calves and that their lungs had fewer lesions of pneumonia than controls after slaughter. Thus, not only was antimicrobial use drastically reduced, but calf health was really improved in the new “outdoor veal calf” system.

**Abstract:**

The “outdoor veal calf” system was developed to encounter the demand for a veal fattening system that allows for reducing antimicrobial use without impairing animal welfare. Management improvements including direct purchase, short transportation, vaccination, three-week quarantine in individual hutches, and open-air housing in small groups in a roofed, straw-bedded paddock with a group hutch were implemented in a prospective intervention study (1905 calves, 19 intervention and 19 control farms, over one year): antimicrobial use was five times lower in "outdoor veal" farms compared to control farms (*p* < 0.001), but it was crucial to ensure that antimicrobial treatment reduction was not associated with decreased animal welfare, i.e., that sick animals were not left untreated. Welfare was assessed monthly on the farms, and organs of 339 calves were examined after slaughter. Cough and nasal discharge were observed significantly (*p* ≤ 0.05) less often in intervention than in control farms, mortality (3.1% vs. 6.3%, *p* = 0.020) and lung lesion prevalence (26% vs. 46%, *p* < 0.001) were lower; no group difference was seen in abomasal lesion prevalence (65% vs. 72%). Thus, besides reduced antimicrobial use, calf health and welfare were improved in "outdoor veal calf" farms in comparison to traditional operations.

## 1. Introduction

Consumers’ attention towards animal welfare has increased in many countries in the last decades [1]. The concept of good welfare conditions presupposes the possibility of living as closely as possible to the original behavior of the species, including no excessive straining of the animals’ adaptability and the absence of health impairments, but also a positive emotional status, especially the absence of stress and fear [2,3].

In contrast to large-scale veal production in other countries, veal calves in Switzerland are often fattened on dairy farms in addition to the production of commercial milk. The male calves of dairy cows and female calves of low genetic value are mostly used for veal production [4]. Veal calves usually enter the fattening unit at the age of four weeks and are housed in groups of 10–60, fed mainly milk or milk replacers, and slaughtered at the age of approximately 160 days, with a weight of about 250 kg [5,6,7,8]. Production of “white” veal is not authorized in Switzerland because ad libitum supply of adequate roughage is prescribed by law [9]; “rosé“ veal is the desired final product as financial deductions may apply at the slaughterhouse if meat color is categorized as "too red". In 2017, 21,865 tons of veal meat (96.6% from domestic production) were consumed in Switzerland [10]. Extra calves were purchased in addition to the calves born on the farm to complete fattening groups in 56% of veal calf operations in a Swiss study [11]. Purchasing calves is associated with an increased risk of metaphylactic antimicrobial treatment and mortality [11]. Metaphylactic treatment consists of treatment of an entire group, including apparently healthy individuals, as soon as clinical signs of disease arise in some of the calves. Such group treatments, mostly with oral preparations, contribute on a large scale to the high antimicrobial use in veal calves, e.g., 84.6% of antimicrobial treatments were given as oral group treatments in a recent study on Swiss veal calves [5]. Similar numbers were also reported in Belgium [12]. As the reduction of antimicrobial use has become a topic of high importance in the veal calf industry [5,12,13], emphasis on the reduction of metaphylactic treatments appears to be a promising strategy.

To reduce antimicrobial use in Swiss veal calf farms, the novel concept “outdoor veal calf” was developed based on the results of recent investigations [5,11]. These studies have shown the main factors associated with increased antimicrobial treatment intensity to include calf purchase, lack of physical examination and quarantine upon arrival at the fattening farm, and shared air space for several groups of calves. Bovine respiratory disease (BRD) was cited as the most common cause of calf death (31.6%) by participating farmers, and metaphylactic antimicrobial treatment was significantly more likely to be applied in herds with a high incidence of BRD [11]. Increased incidence of BRD was also associated with an increased risk of mortality along with large group size (>10 calves) and weight differences (>100 kg) in a group, while vaccination (against BRD) and beef breed were associated with decreased mortality risk [5]. The “outdoor veal calf” concept aims at the elimination of these factors or at least at the mitigation of their effects, by limiting purchase to the direct transport of healthy calves from neighboring farms by the farmer himself, vaccinating calves against BRD upon arrival, and quarantine of all (own and purchased calves) in individual hutches for at least three weeks, followed by outdoor fattening in small groups in a covered, straw-bedded paddock with a group hutch for shelter [8]. The central objective of the project was to investigate the efficacy of the new concept to reduce antimicrobial use by at least 50% in comparison to conventional management. Indeed, the mean treatment incidence on farms operating under the novel system was highly significantly (*p* < 0.001) lower than on farms with conventional management, with values of treatment intensity (TI_DDD_; [14,15]) of 5.9 ± 6.5 vs. 31.5 ± 27.4 days per animal year [8]. However, in order to ensure that the reduction of antimicrobial use would not jeopardize calf welfare, in particular, that sick calves needing treatment would not remain untreated for the purpose of improving antimicrobial use statistics, parameters related to animal health and well-being were also monitored in the frame of the “outdoor veal calf” project. Checklists based on the Welfare Quality Protocol [16] were used to assess calf welfare in a standardized manner and compare animal welfare in the “outdoor veal calf” system with welfare under conventional management. In the present study, the hypothesis that the lower treatment incidence observed in the "outdoor veal calf" farms compared to conventional farms would not occur at the expense of animal welfare, i.e., that indicators of reduced animal welfare such as signs of clinical disease or organ lesions upon slaughter would not be observed more frequently in "outdoor veal" calves than in calves from control farms, was tested.

## 2. Materials and Methods 

All procedures complied with the Swiss legislation on Animal Welfare and Protection (Tierschutzgesetz, Tierschutzverordnung) and were approved by the competent authorities (Committee for Animal Welfare and Protection of the Canton Bern, authorization number BE 71/16).

### 2.1. Study Design and Farm Selection

The "outdoor veal calf" concept and its effect on antimicrobial use, mortality, and daily weight gain in comparison with conventional veal farms have been described in detail elsewhere [8]. The central hypothesis of the "outdoor veal calf" project was that implementing the new concept would reduce antimicrobial use by 50% compared to conventional veal farms. The project was designed as a prospective non-randomized controlled intervention study; sample size calculation to detect a reduction of antimicrobial use of ≥50% between intervention farms (IF) and control farms (CF), with a power of 80% and a confidence level of 95%, indicated that at least 15 farms per group must be included in the study. To ensure a sufficient number of participating farms despite potential drop-offs in the course of the study, 20 farms per group were recruited, of which one per group left the study before its completion. Thus, 19 farms per group participated in the study [8].

The fattening calves in the study farms were kept in addition to a dairy herd, and all herds (IF and CF) belonged to the label organization IP-SUISSE that promotes improved animal welfare and sustainability [17]. Label conditions exceed statutory requirements and include a total area of at least 3.5 m^2^ for calves with a bodyweight under 150 kg and 4.5 m^2^ for animals from 151–300 kg, a bedded area per calf of at least 1.2 m^2^ for calves with a bodyweight under 150 kg, 1.8 m^2^ for calves between 151 and 200 kg, and 2 m^2^ for calves from 200 to 300 kg, and permanent access to an unroofed outdoor area of 1 m^2^ and 1.3 m^2^ for calves weighing less or more than 150 kg, respectively. Group housing or visual contact with other calves for animals housed individually, adequate bedding, and constant roughage and water provision for calves have been mandatory in Switzerland since 2013 [9]. Painful management procedures such as disbudding or castration were not performed on study animals. Between 40 and 80 calves were fattened annually in the study herds, and at least 50% of them had to be purchased from other farms (belonging to any label or conventional operations). The herd managers had given informed written consent to provide access to the veal calf operation as well as to animal and production data over a period of at least 12 months. In addition to the statutory requirements and the IP-SUISSE label’s conditions, only the managers of IF implemented the "outdoor veal calf" system. The managers of IF agreed to perform at least one fattening period with the new system prior to the actual one-year study period. In contrast to IF, the fattening process in CF was not altered.

The IF were enrolled first, CF of similar size, location, and with similar general management practices were accordingly recruited in a second step.

### 2.2. Implementation of the "Outdoor Veal Calf" System

The prerequisites for participation in the “outdoor veal calf” project as an IF included the availability of a sufficient outdoor area for 12 individual calf hutches and two group calf hutches to accommodate 20–30 calves at a time, and a trailer for calf transportation. Furthermore, the farmers had to commit to the strict implementation of the new management system centered mostly on changes in the process of calf purchase as well as in the management and housing of the calves. The cornerstones of the “outdoor veal calf” system are listed below; the system is described elsewhere in detail [8].

#### 2.2.1. Sufficient Colostrum Supply

Farmers were instructed to ask the managers of birth farms from which they purchased fattening calves whether these had received sufficient colostrum supply (i.e., two liters within three hours of birth and another two liters until eight hours after birth), and to buy only calves fulfilling this criterion. For calves born on IF, colostrum supply, as described, was mandatory.

#### 2.2.2. Restrictions for Purchase and Transportation

Calves were purchased exclusively from birth farms in the vicinity to limit transport duration (maximum 30 min). The calves had to be transported directly to the fattening farm without stops and without contact with animals from other farms.

#### 2.2.3. Health Check

Buyers were committed to performing a health check prior to loading, and so to transport only calves without signs of disease.

#### 2.2.4. Vaccination

All calves, i.e., purchased calves and those born on the farm, were vaccinated against BRD upon arrival at the IF or in the second week of life according to the manufacturer’s recommendation, respectively (attenuated live vaccine against bovine respiratory syncytial virus and bovine parainfluenza virus 3, Rispoval^®^ RS + PI3 IntraNasal ad us. Vet, Zoetis Schweiz GmbH, Delémont, Switzerland).

#### 2.2.5. Quarantine

Purchased calves were placed in individual hutches with a small, unroofed paddock (total area 4.1 m^2^) immediately after arrival, they spent at least three weeks there in quarantine. Calves born on the farm and intended for fattening also spent at least three weeks in individual hutches. All calves in individual hutches had visual contact with other calves; however, the hutches had to be set up with a spacing of at least one meter to reduce the risk of transmission of infectious agents.

#### 2.2.6. Small Constant Groups with Limited Weight Differences

**Figure 1:**
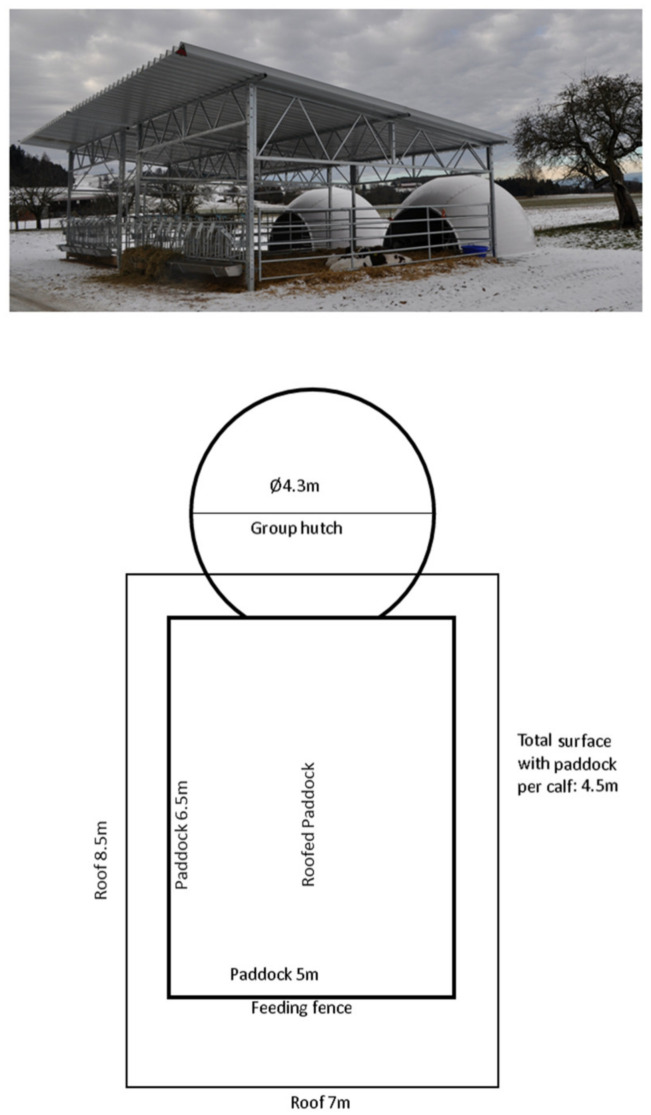
Illustration and dimensions of group hutches in the "outdoor veal calf" system.

After the quarantine period and after reaching the age of at least five weeks, the calves were transferred to group hutches with a roofed paddock (Figure 1).

Groups consisted of a maximum of 10 animals with weight differences ≤50 kg. All calves were moved to the group hutches at the same time as soon as one fattening group was complete; no new calves were added afterward nor exchanged with animals from other fattening groups.

#### 2.2.7. Deep Bedding

Straw bedding had to be abundant (minimal thickness of 30 cm) with a dry top layer. Straw was added regularly depending on weather conditions to maintain adequate bedding quantity and quality. Bedding was provided in hutches as well as in the roofed paddock for group-housed calves.

#### 2.2.8. Cleaning Routine

Farmers were advised to clean and dry the individual and group hutches after use, and to disinfect them with a chlorocresol solution (Neopredisan 135-1^®^, Vital AG, Oberentfelden, Switzerland) according to the manufacturer’s instructions.

#### 2.2.9. Outdoor Housing

Individual and group hutches remained outdoors throughout the year.

#### 2.2.10. Feeding

Milk was fed in buckets with a nipple, with one individual bucket per calf. A mobile heated milk tank with a pump (“milk taxi”, Holm and Laue, Westerrönfeld, Germany) for efficient milk delivery to the calves was provided for each IF. The farmers determined the total amount of milk distributed daily and the number of meals per day (no requirement regarding this point were included in the study protocol). The managers of all IF chose to feed their calves twice daily.

### 2.3. Calf Management on Control Farms

Calf purchase, management, and feeding were not modified on CF; rearing conditions remained as described above under “Study Design and Farm Selection”. An automated milk delivery system was in use on all CF, providing the calves with continuous access to milk.

An overview of the main characteristics of the fattening process in IF and CF is given in Table 1. A detailed description of IF and CF, and of their recruitment for the study, has been given elsewhere [8].

### 2.4. Animal Data

Individual animal data (birth date and date of slaughter or death, gender, breed, date of arrival at the farm—for purchased calves—, date of vaccination, and date of moving to the group hutches) were recorded and linked to the animal’s individual number (ear tag). The information provided by the farmers was collated with the data provided by the Swiss animal movement database (Tierverkehrsdatenbank, TVD, Bern, Switzerland), and any discrepancies were clarified with the farm managers. 

Antimicrobial treatments were recorded in a custom-made booklet containing detailed information about disease duration and treatment results in addition to the standard treatment journal prescribed by Swiss law that includes data about the date, number, and identification of treated calves, the reason for treatment, name, and dosage of the drug, application route, treatment duration, and withdrawal period.

### 2.5. Data Acquisition During Farm Visits

All data were collected by a team of four veterinarians, whereby one investigator conducted 70% of all farm visits and trained the other examiners to ensure consistent data collection.

During the study period from October 2016 to July 2018, both IF and CF were visited monthly. The effective starting time point of the study differed between farms; each farm was observed for at least 12 months. An appointment was made with the farmers for every visit to ensure their presence and thus allow for the exchange of information during the visit. On every visit, the treatment records were checked with the farmers for completeness and accuracy.

The animal welfare assessment was based on the Welfare Quality Protocol [16], with adjustments to the study design and available information. Animal welfare was assessed according to the four welfare principles of “good feeding”, “good housing”, “good health”, and “appropriate behavior”. “Good feeding” included the assessment of body condition, as well as of access to water. In addition to the Welfare Quality protocol, ad libitum provision of roughage, as prescribed by the Swiss legislation [9], was recorded. “Good housing” was assessed by scoring animal cleanliness, presence of wet animals, the slipperiness of the floor, bedding quality and quantity according to a scoring system developed in addition to the Welfare Quality Protocol, as well as recordings of condensation water or mold in the hutches. The scoring criteria are shown in Table 2. “Good health” was assessed as the absence of enlarged subcutaneous bursae, of lameness, skin alterations, bloat or sunken flanks, ocular discharge, signs of BRD, liquid feces and mortality. In addition to the Welfare Quality Protocol, a scoring system for cough and nasal discharge was used as described in Table 2.

Due to practical constraints, no behavioral observations were performed in the frame of the study. For the same reason, the assessment of the fourth and last welfare principle, “appropriate behavior”, was limited to the determination whether lesions due to cross-sucking (reddish, hairless areas on the tail or ear tips) were present or not, as indicators of disturbed calf-specific behavior. These lesions were scored during the last farm visit before slaughter (Table 3). Since health status is a component of welfare according to the Welfare Protocol, the term "welfare" in its further use always includes calf health as well.

#### 2.5.1. General Management Assessment

An extensive questionnaire was completed with the farmers once during the study. Among others, the following important parameters regarding animal welfare were assessed: dam vaccination, birth and colostrum management, care of the neonates, supplementation with iron and selenium, water supply, and cleaning routines.

#### 2.5.2. Monthly Questionnaires

A short questionnaire was completed with the farmers on every monthly visit. Farmers were asked about dead calves and unwanted early slaughter during the previous month, and (if known) the reason thereof. Mortality was defined as death caused by sickness or euthanasia during the observation period. Unwanted early slaughter was defined as slaughter prior to 70 days of fattening [18]. The amount of straw and hay used during the previous month as well as the calves’ consumption of milk and milk powder as estimated by the farmers for individual and group hutches in IF, corresponding to young and older calves in CF as defined below, were recorded. The observations relative to animal welfare during farm visits included parameters of management, calf health, and other welfare indicators, as described in Table 2. Because of the importance of BRD in veal fattening farms, the parameters “cough” and “nasal discharge” were evaluated in detail, i.e., at the calf group level. In IF, these parameters were assessed separately for young calves in individual hutches and for older calves in group hutches, whereas in CF the calves were classified in a category corresponding to "individual hutches" based on their age (≤ five weeks) as young calves were already kept in groups from the beginning of the fattening period; older calves were accordingly assigned to a category corresponding to "group hutches". For every group and clinical sign, the percentage of all calves with signs of BRD was calculated, resulting in four values in % (cough in individual hutches (CI), cough in group hutches (CG), nasal discharge in individual hutches (NI), and nasal discharge in group hutches (NG)). The severity of the observed clinical signs was recorded at the group level as well, whereby the attributed score represented the mean degree of severity for all calves showing the corresponding clinical sign in a group.

#### 2.5.3. Individual Finishing Checklists

On the last visit before slaughter, the health status of every calf was assessed individually according to a checklist developed based on the Welfare Quality Protocol [16]. The recorded parameters and the scoring system are described in Table 3.

### 2.6. Assessment of Lung and Abomasal Lesions at Slaughter

Farmers were requested to advise the study team when calves were sent to slaughter so that the organs of a subset of them could be examined. These calves had to be slaughtered in one of three slaughterhouses where the study team was allowed to collect samples. After slaughter, the lungs and the abomasum were marked with an individual number related to the ear tag number of the calf and retrieved from the slaughter line for examination. The lungs were inspected visually from both sides and palpated. Lesions suggestive of pneumonia and pleural adhesions were recorded. The abomasa were separated from the rest of the digestive tract, and a 13 cm section was dissected from the pyloric area and cut open longitudinally. After rinsing to remove abomasal contents, lesions of the mucous membrane were recorded if present. Both lungs and abomasa were assessed according to a scoring system based on the Welfare Quality Protocol [16], as shown in Table 4.

All organ examinations were performed by the same investigator who was blinded regarding the provenance of the calves, i.e., who did not know from which farm group (IF or CF) the calves originated. Organs were photographed in the slaughterhouse, and attribution of the scores was validated in a second step by a board-certified pathologist who was also blinded as to the origin of the specimens.

### 2.7. Data Analyses

All data were entered into the Access database management system (Access 2016, Microsoft^®^, Redmond, WA, USA) and prepared for analysis with the software Excel (Excel 2016, Microsoft^®^, Redmond, WA, USA). Statistical analyses were performed with the statistical software NCSS 12 (Kaysville, UT, USA).

Quantitative data describing the study population were tested for normality with Shapiro-Wilk W tests; the unit of analysis was the farm. Depending on data distribution, Two-Sample *T*-tests or Mann-Whitney U tests were used to test for differences between IF and CF.

For data recorded during the last pre-slaughter visit and at slaughter, the level of analysis was the individual animal. For data recorded during the monthly farm visits, the level of analysis was the animal groups present during the visit. The results were categorical, ordinal, or consisted of prevalence. We assumed, therefore, non-normal distribution and applied nonparametric tests. Parameters for which only a few (<five) observations were available were excluded from further analyses, or, in the case of scores, unfrequently observed scores (e.g., the highest one) were merged with the next category. The prevalence of calf losses, cough, nasal discharge, and the mean abomasal lesion score were tested for differences between the intervention and control groups using the Mann-Whitney-U test. Chi-Square tests were used for categorical data and scores with less than five categories (all other observations). Due to missing values in the monthly questionnaires, the total number of recorded variables available for analysis was not always the same; the number of available observations is given in Table 4, Table 5 and Table 6. We checked for a possible influence of season and visit number on the data recorded on monthly visits using Kruskal-Wallis and Chi-Square tests.

## 3. Results

### 3.1. Study Population

A total of 900 calves from IF and 1005 from CF was included in the study. The limited group size defined for IF resulted in a lower number of fattened calves per year as compared to CF (41.0 ± 11.3 vs. 53.6 ± 11.8 calves per year; *p* = 0.002). The observation period in IF was accordingly longer (432 ± 58.9 days vs. 349 ± 4.0; *p* = 0.002) to obtain comparable calf numbers. Other population characteristics such as proportions of sex and breed, as well as purchase rate and age at purchase, did not differ significantly between IF and CF [8].

### 3.2. Results of General Management Assessment

Results of the general management assessment revealed a similar management quality of IF and CF for parameters which were not prescribed in the “outdoor veal calf” system (e.g., birth management and care of the neonates, dam vaccination against calf scours, supplementation of calves with iron and selenium, water supply and cleaning routines). Regarding parameters defined in the “outdoor veal calf” system, colostrum management in CF was as good as in IF, whereas only a few CF vaccinated the calves against BRD. Individual quarantine upon arrival was practiced only in three of the 19 CF; purchased animals (from different origins) were kept together in quarantine groups after arrival on five farms. All IF managers followed the advice to clean out the hutches after each use; ten farmers disinfected the hutches according to the instructions. No CF performed cleaning out of all facilities after each use; individual pens or hutches were cleaned out on three farms. We used this assessment to generate an overview of the structure of the participating farms and did not analyze it further, statistically.

### 3.3. Results from the Monthly Questionnaires

A total of 306 and 229 monthly questionnaires were filled in the 19 IF and 19 CF, respectively, resulting in a median value of 16 (range: 13–20; interquartile range (IQR): 14–18) questionnaires per IF and 12 (range: 11–14; IQR: 12–12) per CF. The difference in the number of completed questionnaires resulted from the longer observation period in IF. We found no significant association of visit number, i.e., of the time point in the course of the project, with the results of the monthly questionnaires. Distribution of visits over the four seasons showed no difference between IF and CF (*p* = 0.26). For this reason, no effect of seasonal variability on the validity of group comparison was expected. 

Information on calf losses, i.e., mortality and early slaughter, given by the farmers, was compared and completed with data from the TVD. Calf losses were significantly (*p* = 0.02) lower in IF than in CF, with a mean (± SD) of 3.1 (±2.3) % in IF vs. 6.3 (±4.9) % in CF. The causes of death reported by the farmers included BRD (IF: 2, CF: 21), diarrhea (IF: 4, CF: 2), poor growth (IF: 2, CF: 4), and unknown reasons (IF: 18, CF: 40). Necropsy was not performed systematically.

The results of the group assessment are listed in Table 5.

The floor was less slippery, with less liquid feces, and the animals were cleaner in IF compared to CF, whereas amount and cleanliness of the bedding and provision of roughage was not significantly different in the two groups. Prevalence (in %) of following signs of BRD during individual herd visits was significantly lower in IF than in CF: cough in individual hutches (IF: median = 0, IQR = 0–0; CF: median = 0, IQR = 0–11.1, *p* = 0.009), cough in group hutches (IF: median = 0, IQR = 0–10.5; CF: median = 9.1, IQR = 0–20.0, *p* < 0.001) and nasal discharge in group hutches (IF: median = 5.3, IQR = 0–22.2; CF: median = 16.2, IQR = 0–33.3, *p* < 0.001). Prevalence of nasal discharge in individual hutches did not differ significantly between groups (IF: median = 0, IQR = 0–20.0; CF: median = 0, IQR = 0–25.8, *p* = 0.057). The severity of cough and nasal discharge was not further analyzed.

### 3.4. Results of Individual Finishing Checklists

A total of 783 calves (87%) in IF and 779 (76%) in CF were examined individually at the end of the fattening period. The remaining calves were sent to slaughter without previous notice before the next farm visit. No difference was observed between IF and CF in the results of the pre-slaughter examination; detailed results are given in Table 6.

### 3.5. Results of Lung and Abomasal Lesions Assessment at Slaughter

The organs of 339 calves (IF: 168, CF: 171, from 17 farms each) were inspected after slaughter. A total of 327 abomasa and 332 pairs of lungs were available for inspection. The missing organs were lost or damaged during the slaughter process. Higher scores for lung lesions and pleural adhesions were observed in CF than in IF calves, whereas no differences were observed in abomasal lesion scores (Table 7).

## 4. Discussion

The goal of the present study was to compare calf welfare in the “outdoor veal calf” system and in conventionally managed Swiss veal farms. While the main objective of the overall “outdoor veal calf” project was to test the hypothesis that antimicrobial use can be reduced by at least 50% through the implementation of a novel management and housing concept, ensuring that decreased treatment intensity would not be associated with reduced animal welfare was a central point of its evaluation. The present results confirm that the reduction of antimicrobial treatments in “outdoor veal calf” farms was not achieved at the cost of animal welfare, i.e., as a consequence of inadequate therapy for BRD, the main indication for antimicrobial treatments in veal calves [7,19]. Indeed, significantly fewer signs of BRD in live animals and fewer lung lesions at slaughter were observed in IF than in CF calves.

The evaluation of the effects of single management parameters (e.g., housing or vaccination) in the frame of field studies is complicated by the fact that their effect can be mitigated by overall good management practices [20,21]. The evaluation of single measures to decrease the need for treatment and improve animal welfare was not in the focus of our study. Instead, the overall concept of "outdoor veal calf" comprising measures to minimize the effects of various risk factors associated with increased treatment intensity and mortality was evaluated. 

A strong association of calf purchase with increased antimicrobial use and increased mortality has been repeatedly demonstrated [5,7,11]. Limiting the duration of transport, performing a health check prior to purchase and refusing sick animals, as well as implementing quarantine in individual hutches after arrival at the fattening farms, were included in the "outdoor veal calf" concept in order to minimize the effects of calf purchase. Establishing a close network between calf providers and buyers allowed on the one hand for decreasing the transportation time, thus reducing the stress and the risk of dehydration associated with transports over long distances [22,23]. On the other hand, direct purchase also increased social pressure on the sellers and motivated them to offer only healthy, well-conditioned calves for purchase. Indeed, IF farmer reported that no sick calves were offered for purchase or had to be rejected during the study. The benefits of sufficient colostrum supply [22,24] are well known; an adequate supply of colostrum was mandatory for calves born on IF and had to be confirmed by the seller for purchased calves. Most of the CF had good colostrum management for their own calves as well, but no information on the colostrum supply of purchased calves was available.

Vaccination against BRD should take place, preferably on the birth farms, long enough before transport to generate sufficient immunity prior to challenge with pathogens derived from calves from other farms [25]. Calves in IF were mostly vaccinated upon arrival in the fattening operation, but the time until adequate vaccine protection developed was bridged through the mandatory quarantine period of at least three weeks. A quarantine timespan of three weeks has been reported to be effective [26].

The most common cause of antimicrobial treatment in Swiss veal calves is BRD, however, a large part of these treatments are applied metaphylactically [11], i.e., some of the calves are treated without being clinically diseased. Signs of BRD were regularly observed in study farms during monthly visits. The fact that a significantly lower prevalence was found in IF than in CF for every respiratory parameter, except for nasal discharge in young calves (*p* = 0.057), suggests that the implementation of the “outdoor veal calf” system is effective in preventing BRD. These findings support a holistic approach to BRD prevention, as suggested by others [5,27,28]. The prevalence of respiratory signs encountered in our study is comparable to previous reports. In a European study in which respiratory signs were assessed in a similar manner and prophylaxis (antimicrobials, antiparasitic drugs, or vaccination) was used in a standardized manner, BRD prevalence was situated between the values of IF and CF for cough and nasal discharge on a group level with <7% [29]. A higher mean prevalence of 14.3% of respiratory disease was reported for pre-weaned dairy calves in US farms, which is comparable to the values observed in CF, but only animals showing ≥ two respiratory signs were counted as diseased in that study [30]. The same authors reported an increase in the prevalence of respiratory diseases after the age of four weeks, with a peak at seven weeks of age, followed by a decrease in prevalence. The distribution in both groups in our study was similar, as prevalence in older calves (≥five weeks) was higher than in younger ones, and almost no signs of BRD were recorded at the last visit before slaughter.

The significantly higher counts of lesions indicative of pneumonia found in CF calves at slaughter compared to IF calves underline the positive effect of the “outdoor veal calf“ concept on the respiratory system of fattening calves. In accordance with findings of others [29], we found a distinctly higher prevalence of lung lesions at slaughter (26% lung lesions and 3% of pleural adhesions in IF, and 46% and 11% in CF, respectively) than of previously observed clinical signs of BRD, suggesting that even mild cases of pneumonia can cause permanent lung damage. Alternatively, the prevalence of disease may have been underestimated in this study, as clinical signs were recorded only by observing the animals without individual clinical examination (e.g., lung auscultation or thoracic ultrasound).

Perforating ulcers of the abomasum are an important cause of mortality in calves. Bähler et al. [18] found perforating abomasal ulcers to be the cause of 22% of deaths in Swiss veal calves. The occurrence of abomasal pyloric lesions found in our study (65% (IF)–72% (CF) of examined abomasa) is similar to the findings of other authors [31,32]. Abomasal ulcers have a multifactorial etiology, whereby stress and feeding techniques play important roles [31,33]. A study in foals, where, in contrast to calves, a survey in living animals can be performed via endoscopy, showed gastric lesion prevalences between 47 and 57% in foals without signs of gastric disease. Previous disease was associated with a higher prevalence of gastric lesions [34]. Foals examined before and after weaning had a higher prevalence of gastric lesions after weaning, suggesting that the stressful event of weaning promotes lesion formation [35,36]. In the present study, the prevalence of abomasal lesions, assessed as indicators of stress, did not differ between IF and CF. As management differed in several points between the two systems, it is not possible to determine the influence of individual factors on the development of abomasal lesions, i.e., whether the prevalence of abomasal lesions was not affected by the implementation of the “outdoor veal calf” system or whether positive and negative effects counterbalanced each other. Several authors postulated that fewer meals per day, resulting in a higher volume fed per meal, represent a risk factor for pyloric abomasal lesions [31,32,37]. In our study, all IF calves were fed twice daily, whereas automatic feeders allowing more meals per day were present in all CF. The impact of abomasal lesions on calf welfare is difficult to quantify as a diagnosis in vivo is not possible in ruminants (except for bleeding and perforating ulcers) [33]. Nevertheless, gastric lesions are known to be painful in monogastric species, and perforating ulcers are an important reason for death in calves [31,33,38], and an impact on animal welfare must, therefore, be considered.

We recorded the prevalence of diarrhea on the one hand at the group level, whereby only the presence of liquid manure on the floor was recorded during the monthly farm visits, so the number of calves that actually had diarrhea was not taken into account. Nevertheless, as the same method was used for both IF and CF, a comparison between the two groups of the study is granted. As the presence of liquid manure on the ground is easier to detect on bare floors than in deep bedding, this feature may have been underestimated more often in IF for older calves as deep bedding was always present. However, as the group difference is large (7% in IF vs. 20% in CF), a truly higher prevalence of diarrhea in CF must be suspected. On the other hand, we recorded the occurrence of liquid manure at the single calf level at the individual finishing assessment and found the same prevalence in IF as in CF (33%). A comparison of the two results regarding the occurrence of liquid feces (at the group vs. individual level) is difficult due to the different assessment strategies. Determination of the causes for liquid feces was beyond the scope of the study, especially because the calves in both groups were not necessarily fed milk in a comparable way (twice a day feeding with the “milk taxi” in IF vs. automated ad libitum feeding in CF). Furthermore, the composition of the liquid diet also varied among farms. The lower prevalence of diarrhea observed in IF during monthly visits suggests that the risk of pathogen buildup in deep bedding [39] could be mitigated by thorough cleaning after each fattening group. Even though the facilities were not disinfected in all farms after use, as intended in the “outdoor veal calf” system, the used bedding was completely removed and the facilities cleaned in all IF. A positive effect on the prevalence of diarrhea of all-in all-out systems and subsequent cleaning of the facilities after the departure of the animals is well established [19,40].

In accordance with the lower prevalence of diseases recorded in IF, the overall mortality was also lower than in CF. Mortality in IF was similar to the results of other Swiss investigations [7,11,18] and one recent work from Belgium [41], whereas CF had higher mortality rates, which were more comparable to other results from Belgium [42] and Canada [20,43]. This high mortality rate is possibly due to the fact that the date of death of every calf was checked in the TVD in our study, i.e., early death was recorded more accurately than in other studies where mortality rates were reported retrospectively by farmers. The managers of CF reported distinctly more cases of calf losses due to BRD than those of IF, which is consistent with the results of the monthly visits and the prevalence of lung lesions found at slaughter.

The health benefits of individual housing early in a veal calf’s life has been well documented [29,44]. However, an optimal duration of the individual housing period is difficult to define and may vary depending on the management system. For older calves, the positive effects of group housing are assumed to prevail over the inherent challenges [29]. In addition to production and health parameters such as dry matter intake and weight gain [45,46], group housing meets the calves’ need for social interaction and thus contributes to better welfare [47,48,49]. Group housing is prescribed by Swiss law for calves >14 days old, except for those kept in individual hutches with visual contact with others [9]; thus, both IF and CF housed the older calves in groups. Space allowance per IF calf at full occupancy of the group hutches was 1.35 m^2^ in the hutch plus 3.25 m^2^ in the roofed and bedded paddock. Calves in CF had the same area at their disposal according to the label requirements of IP-SUISSE, which is rather generous in comparison to other systems [38]. The superior results in health parameters in IF may not be due to group housing itself or available space per calf, but rather to the defined group characteristics in the “outdoor veal calf system”. A group size ≤10 animals was found to be beneficial for calf health [50] and to reduce antimicrobial treatment incidence [11]. Weight differences >100 kg were associated with increased mortality [5]; on this account, the formation of groups of calves of similar age and weight was included in the "outdoor veal calf" concept. Group housing can, however, also be a risk factor for disease, as pathogens can spread more easily due to close contact among animals [26,40]. No new calves were added to an existing fattening group in IF; the possible introduction of new pathogens was limited by these means. Our results suggest that not group housing itself, but various features of the groups play an important role and can profoundly influence the success of a housing system.

The strict outdoor housing of the calves throughout the year was a delicate issue in IF, as calves are known to be sensitive to draughts [5,7,18]. However, the reduced treatment incidence, the reduced occurrence of clinical signs of BRD, and the reduced prevalence of lung lesions at slaughter indicate that IF calves received sufficient protection against cold temperature and wind in the hutches. Protection from direct solar radiation was provided by the roof, which covered the hutch as well as the paddock. The thermoneutral zone for calves is estimated to be between 0 and 25 °C for calves during the first month of life and −5 to 22 °C for veal calves, and this range can be extended if the calf is acclimatized to higher or lower temperatures [51]. The environmental temperatures measured on a meteorological station in the study region in 2017 ranged from −10 to 30 °C [52]. Thermal stress in cold temperatures can be counterbalanced by providing a sufficient amount of dry bedding and a shelter as in our study setting [30,51]. Beneficial effects of straw bedding arise only if it is clean and dry, and allows nesting [30], which was mostly the case in IF. The amount and quality of bedding used in IF and CF did not differ, but in contrast to IF where the entire paddock was bedded with at least 30 cm of straw, CF barns included a non-bedded, non-roofed paddock for the veal calves. This difference explains the different characters of floor slipperiness for IF and CF and probably also the difference in animal cleanliness. Even though calves were less clean in CF than in IF (84% vs. 92% of clean animals, respectively), these values suggest a good stockmanship in all participating farms, as other authors reported detrimental effects of deep bedding on calf cleanliness [53,54,55]. Deep bedding also provides protection from bursitis and lameness [55]; these pathologies were very rare in IF and in CF.

Comparison between IF and CF under the conditions of our study revealed a high level of animal welfare in both systems. All participating veal calf farmers produced according to a quality label program and may represent a population of above-average herd managers. A selection bias can thus be suspected as forward-thinking farmers may be more likely to participate in a scientific study; however, this applied to both groups. It cannot be excluded that IF farmers were more motivated than the farmers of CF, as they were willing to thoroughly change their routines to participate in the study; however, the commitment of CF farmers was also high, as they agreed to provide detailed data and to participate in monthly herd visits over an entire year. We did not encounter major differences between groups in the individual management quality of the participating farms. Some of the management improvements included in the “outdoor veal calf“ system were also practiced in CF. Therefore, we assume that the differences observed in calf welfare between CF and IF are based on the consistent implementation of the entire “outdoor veal calf” system and not on the effects of isolated measures towards improved calf management.

Finally, economic analysis, including costs of housing, feeding, labor as well as disease and treatment costs, will be needed to assess the profitability of the "outdoor veal calf" system. These results, which will be reported elsewhere, will eventually determine the sustainability of the new system for veal farmers and show whether specific incentives should be recommended to support the implementation of a system that allows for a drastic reduction of antimicrobial use and improvement of animal welfare in Swiss veal calf operations.

## 5. Conclusions

Veal calf production according to the “outdoor veal calf” system was shown to improve animal welfare, in addition to strongly reducing the need for antimicrobial treatments, compared to conventional label farming. Our results underline the importance of a holistic approach for the prevention of diseases with a multifactorial etiology such as respiratory disease. The “outdoor veal calf system” can be implemented in many farms with relatively little effort and investment under Swiss farming conditions, and provides, therefore, a pragmatic approach to address the high consumption of antimicrobials in the veal calf industry. Strict outdoor housing, even during the cold and warm seasons, did not impair the welfare of calves, and the new management system was associated with improved calf health. Similar production systems adapted to other settings than those encountered in Switzerland can be developed elsewhere, based on the information provided in this study.

## Figures and Tables

**Table 1:** Characteristics of the main interventions on intervention farms (IF) implementing the novel system “outdoor veal calf” and corresponding procedures on control farms (CF).

Farm Type Intervention	Intervention Farms (IF) “Outdoor Veal Calf”	Control Farms (CF)
Implementation of the “outdoor veal calf” system	Implementation of three major interventions: (a) direct purchase, (b) quarantine and vaccination, and (c) strict outdoor housing with a sheltered paddock	No implementation of the novel system, no intervention (no changes in the farm routines); fattening according to IP-SUISSE standards
Sufficient colostrum supply	At least 2 × 2 L of colostrum within 8 h of birth for calves born on the farm and purchased calves (to be confirmed by the seller prior to purchase)	No specific regulations given in the frame of the study
Restrictions for purchase and transportation	≥50% purchased calves over the study period; direct purchase by the farmer (no purchase through livestock dealers) from close farms (transportation time ≤30 min), only direct transport in a private trailer, transport of ≤10 calves at a time, no mixing of calves from different birth farms	≥50% purchased calves over the study period; no further requirements or restrictions regarding purchase and transport
Health check prior to purchase and transport	Mandatory health check of purchased calves prior to transport, purchase of calves with signs of disease not authorized	No specific regulations given in the frame of the study
Vaccination	Vaccination of all calves against bovine respiratory disease mandatory	No specific regulations given in the frame of the study
Quarantine	Quarantine for all calves (home-born and purchased) in individual hutches for at least three weeks mandatory	No specific regulations given in the frame of the study
Small constant groups with limited weight differences	≤10 calves of similar estimated weight grouped after quarantine	No specific regulations given in the frame of the study
Deep bedding	Deep straw bedding (≥30 cm thickness) in individual and group hutches, and in the roofed outdoor pen of group hutches	No specific regulations given in the frame of the study; no bedding in unroofed outdoor paddock (IP-SUISSE guidelines)
Cleaning routines	Hutch/pen cleaning and disinfection after each use	No specific regulations given in the frame of the study
Outdoor housing	Strict outdoor housing for all calves during the entire fattening period	No specific regulations given in the frame of the study (constant access to a non-roofed outdoor pen prescribed by IP-SUISSE)
Feeding	Semi-automated feeding with manually controlled milk-delivering robot twice daily	No specific regulations given in the frame of the study (automated ad libitum feeding in all CF)

**Table 2:** Scoring system for indicators of calf welfare during monthly farm visits.

Parameter	Score			
	0	1	2	3
Amount of bedding	At least 30 cm of bedding material	<30 cm bedding, floor not visible	Almost no bedding, floor visible	
Cleanliness of the bedding	Dry and clean	Some soiled bedding visible	Entire bedding soiled and wet	
Slipperiness of the floor	No or limited slipperiness	Free movement of calves impaired	Difficult to stand and move on the floor	
Condensation water in hutches/stables	None	Condensation water visible under the roof		
Mold in hutches/stables	None	Mold visible on the inside of the roof		
Signs of diarrhea	No liquid manure on the ground	Liquid manure on the ground		
Cleanliness of the animals	More than 2/3 of the animals are clean ^1^	Between 1/3 and 2/3 of the animals are clean ^1^	Less than 1/3 of the animals are clean ^1^	
Cough ^2^	No cough	Superficial, dry cough ^3^	Loud, repeated, not productive cough ^3^	Rattling, productive cough ^3^
Nasal discharge ^2^	No or small amount of serous discharge	Mucous discharge ^3^	Muco-purulent discharge ^3^	Heavy, purulent discharge ^3^

^1^ Clean ≤25% covered with plaques and <50% of the body covered with liquid dirt. ^2^ These parameters were evaluated at the calf group level, for young calves (in individual hutches in IF and calves ≤ five weeks old in CF), and older calves (in group hutches in IF and > five weeks of age in CF) separately, during the course of a farm visit lasting approximately 45 min. For every group and clinical sign, a percentage rate of calves with signs of disease was calculated. ^3^ The score represents the mean degree of severity for all calves in a group showing the respective clinical sign.

**Table 3:** Scoring system for individual calf examination at the last farm visit before slaughter.

Parameter	Score		
	0	1	2
Body condition	Normal	Visibly thinner than calves of the same age	Severely thinner than calves of the same age
Calf cleanliness	Clean: <25% covered with plaques and <50% of the body covered with liquid dirt	Dirty: >25% covered with plaques or >50% of the body covered with liquid dirt or both	
Signs of heat stress	Dry haircoat on the back	Wet haircoat on the back	
Alopecia on the neck ^1^	None	Present	
Damages to the hair coat ^2^	None	Present	
Lesions due to cross-sucking	None	Reddish, hairless areas on tail or ear tips	
Repeated cough ^3^	No or only singular cough	Frequent, repeated cough	
Forced breathing	None	Difficult or laboredbreathing	
Lameness, swellings of the limbs	None	Lameness or swelling visible	
Presence of liquid feces	None	Liquid feces or perianal region soiled with liquid manure	
Alopecia of the perianal region ^4^	None	Present	
Abdominal shape	Normal	Sunken flanks	Bloat
Ocular discharge	None	Present	

^1^ Technopathy due to an inappropriate feeding fence. ^2^ Areas of alopecia other than on the neck and lesions due to trichophytosis. ^3^ During the course of a farm visit lasting approximately 45 min. ^4^ As an indicator of ongoing or past episodes of diarrhea.

**Table 4:** Scoring system for lung, pleural and abomasal lesions at slaughter.

Lesions	Score			
	0	1	2	3
Lung lesions ^1^	None, normal color and texture	One or more spots of grey-red discoloration on the cranial or middle lobe or both	Several spots of grey-red discoloration on the entire lung or one extended area not including the caudal lobe	Grey-red discoloration on the entire lung, including the caudal lobe, or presence of abscesses
Pleural adhesions	No adhesions, all lung lobes can be separated	Lobes are attached together or to adjacent tissues		
Abomasal lesion size ^2^	No visible lesions	Lesion <0.5 cm^2^	Lesion 0.5–1 cm^2^	Lesion >1 cm^2^
Number of abomasal lesions ^2,3^	N/A	Number of size one lesions	Number of size two lesions	Number of size three lesions

^1^ The highest score observed was recorded. ^2^ An overall abomasal lesion score was calculated according to the Welfare Quality Protocol as the sum of the (number of size 1 lesions × 1), (number of size 2 lesions × 2), and (number of size 3 lesions × 3), resulting in a possible range of values between 0 and 24. ^3^ The number of lesions per score was counted, with all lesions counts of 4 and more assigned to a single category, 4+.

**Table 5:** Comparison of calf welfare indicators at the group level in intervention and control farms evaluated during monthly visits (306 and 229 farm visits, respectively) using Chi-Square tests.

Parameter	Group	n ^1^	Score (%)			*p*-Value
			0	1	2	
Amount of bedding	Intervention	287	74	21	6	0.41
	Control	208	71	20	9	
Cleanliness of bedding	Intervention	283	72	22	6	0.54
	Control	210	70	22	9	
Provision of hay	Intervention	283	23	77	N/A	0.25
	Control	203	28	72	N/A	
Signs of diarrhea on the ground	Intervention	305	93	7	N/A	<0.001
	Control	206	80	20	N/A	
Slipperiness of the floor	Intervention	223	96	4	1	<0.001 ^2^
	Control	150	65	22	13	
Cleanliness of the animals	Intervention	295	92	5	3	0.02
	Control	222	84	11	5	
Condensation water in the hutches or stables	Intervention	274	97	3	N/A	N/A^3^
	Control	185	97	3	N/A	
Mold in the hutches or stables	Intervention	727	100	0	N/A	N/A^3^
	Control	185	96	4	N/A	

^1^ n = number of observations. ^2^ Original score 2 was merged with score 1 for statistical analysis due to low observation counts. ^3^ N/A, not applicable, i.e., not observed or not analyzed statistically due to the low number of observations.

**Table 6:** Comparison of health and welfare scores at an individual animal level in intervention and control farms (783 and 779 calves, respectively) during the last farm visit before slaughter using Chi-Square tests.

Parameter	Group	n ^1^	Score (%)		*p*-Value
			0	1	
Body condition	Intervention	783	98	2 ^7^	0.36
	Control	779	98	2 ^7^	
Cleanliness	Intervention	783	100	0	0.25
	Control	779	99	1	
Signs of heat stress	Intervention	783	100	0	N/A ^6^
	Control	779	100	0	
Alopecia on the neck ^2^	Intervention	783	99	1	0.34
	Control	779	96	4	
Damages of the hair coat ^3^	Intervention	783	97	3	0.44
	Control	779	88	12	
Lesions due to cross-sucking	Intervention	783	100	0	N/A ^6^
	Control	779	100	0	
Repeated cough ^4^	Intervention	783	99	1	0.59
	Control	779	99	1	
Forced breathing	Intervention	783	100	0	N/A ^6^
	Control	779	100	0	
Lameness or limb swellings	Intervention	783	100	0	N/A ^6^
	Control	779	99	1	
Presence of diarrhea	Intervention	783	67	33	0.34
	Control	779	67	33	
Alopecia of the perianal region ^5^	Intervention	783	98	2	0.46
	Control	779	99	1	
Abnormal abdominal shape	Intervention	783	100	0 ^8^	N/A ^6^
	Control	779	100	0 ^8^	
Ocular discharge	Intervention	783	94	6	0.29
	Control	779	91	9	

^1^ n = number of observations. ^2^ Technopathy due to an inappropriate feeding fence. ^3^ Areas of alopecia other than on the neck and lesions due to trichophytosis. ^4^ During the course of a farm visit lasting approximately 45 min. ^5^ As an indicator of ongoing or past episodes of diarrhea. ^6^ N/A, not applicable, i.e., not analyzed statistically due to the low number of observations (< five). ^7^ Original score 2 was merged with score 1 due to observation counts of 0 or 1. ^8^ Orginal scores 1 “sunken” and 2 “bloat” were merged to one score 1 “abnormal abdominal shape”.

**Table 7:** Comparison of organ lesions at slaughter between intervention and control farms (168 and 171 calves, respectively), using Chi-Square ^1^ or Mann-Whitney-U tests ^2^.

Parameter	Group	n ^3^		Score (%)			*p*-Value
				0	1	2	
Lung lesions ^1^	Intervention	164		74	18	7 ^4^	<0.001
	Control	168		54	24	21 ^4^	
Pleural adhesions ^1^	Intervention	164		97	3	N/A ^5^	<0.001
	Control	168		89	11	N/A ^5^	
				No	Yes		
Presence of abomasal lesions ^1^	Intervention	165		35	65		0.22
	Control	162		28	72		
		Median	Min	Max	Interquartile range		
Abomasal lesion score ^2,6^	Intervention	2	0	24	0–4		0.13
	Control	2.5	0	12	0–4		

^1^ Analyzed using Chi-Square test. ^2^ Analyzed using Mann-Whitney-U test. ^3^ n = number of observations. ^4^ Original score 3 was merged with score 2 due to observation counts between 0 and 1. ^5^ N/A, not applicable; pleural adhesions were recorded as present or not. ^6^ For a definition of abomasal lesion score, see Table 4. The level of significance was set at *p* ≤ 0.05.
